# STUCK IN RUINS, OR UP AND COMING? THE SHIFTING GEOGRAPHY OF URBAN PUBLIC HEALTH RESEARCH IN KISUMU, KENYA

**DOI:** 10.1017/S0001972013000442

**Published:** 2013-11

**Authors:** P. Wenzel Geissler

## Abstract

Since the Second World War, the Kenyan city of Kisumu has been an important site of medical research and public health interventions – on malaria and other vector-borne diseases, and lately on HIV and related infections. This article compares the work and lives of two generations of local workers in public health research, each central to science in the city at their time: staff of the Ministry of Health's Division of Vector Borne Disease (DVBD) in the decades after independence, and temporary employees of the Kenyan Medical Research Institute (KEMRI) in its collaboration with the US government's Centers for Disease Control and Prevention (CDC) in the early twenty-first century. Against the backdrop of changes to the city, which stagnated during the 1970s and 1980s, became an epicentre of the East African AIDS epidemic, and underwent an economic boom of sorts from the late 1990s – at least partly driven by HIV research and intervention programmes – the article examines the spaces and movements of health research workers, and their experience of the city in time. The now elderly DVBD workers' accounts are pervaded by memories of anticipated progress and the convergence of life and work in the civic wholes of nation and city; by chagrin about decay; and by nostalgia for lost hopes. Today's young KEMRI/CDC workers' short-term contracts, and the fragmented city they inhabit and study, make for less bounded and predictable spaces and temporalities. Their urban lives and work take shape between remainders and remembrances of past projects, the exhaustion of everyday struggles to make a living and a meaningful life, and the search for new forms of urban order and civic purpose.

## RÉSUMÉ

Depuis la Seconde Guerre mondiale, la ville kenyane de Kisumu est un haut lieu de la recherche médicale et des interventions en santé publique, notamment pour le paludisme et autres maladies transmises par un vecteur, et plus récemment le VIH et infections associées. Cet article compare le travail et l'existence de deux générations de travailleurs locaux impliqués dans la recherche en santé publique, chacune occupant à son époque un rôle central pour la science dans la ville : des employés du DVBD (Division of Vector Borne Disease, département du ministère de la Santé responsable des maladies transmises par vecteur) durant les décennies qui ont suivi l'indépendance, et des employés temporaires de l'institut de recherche médicale KEMRI (Kenyan Medical Research Institute) dans le cadre de sa collaboration avec le CDC (Centers for Disease Control and Prevention, département américain responsable du contrôle et de la prévention des maladies) au début du vingt-et-unième siècle. Avec en toile de fonds les changements intervenus dans la ville qui a connu une période de stagnation dans les années 1970 et 1980 avant de devenir un épicentre de l’épidémie de SIDA en Afrique de l'Est et de connaître un certain essor économique à partir de la fin des années 1990 (insufflé, du moins en partie, par les programmes d'intervention et de recherche sur le VIH), l'article examine les espaces et les mouvements des travailleurs de recherche en santé, et leur expérience de la ville. Les récits des employés du DVBD, aujourd'hui âgés, sont imprégnés de souvenirs de progrès attendu et de convergence de la vie et du travail dans la totalité civique de la nation et de la ville, de dépit face au déclin, et de nostalgie d'espoirs perdus. Les contrats à court terme des jeunes employés du KEMRI/CDC, et la ville fragmentée dans laquelle ils habitent et étudient aujourd'hui, se traduisent par des espaces et des temporalités moins délimités et prévisibles. Leur existence et leur travail dans la ville prennent forme entre ce qu'il reste des projets passés et de ce que l'on en retient, l’épuisement d'une lutte quotidienne pour la subsistance et une existence décente, et la quête de nouvelles formes d'ordre urbain et de sens civique.

This article traces the shifting geography of ‘street-level’ science work in the East African city of Kisumu by comparing two generations of local workers in public health research – one working in the present, the other remembering work and the city thirty or more years ago. Contributing to literature on the interaction of science and place – how science has shaped the city (for example, Packard 1990), how the city can be read as an ‘archive’ of past inscriptions (Quayson 2010), or how it is made part of producing and legitimizing scientific knowledge (see, for example, Gieryn 2006) – the article focuses on the engagement of place and work in the envisioning, and remembrance, of civic projects: the city as ‘man's method of expression’ in pursuit of association and well-being, a layered work of pasts and futures (Mumford 1961); or as an ensemble of movements and work that calls forth the polis, a material ‘space of appearance’ through which one's participation (or not) in a larger social entity takes form (see Arendt 1951, 1958: 199).^1^ One's habitation and circulation in a city reflect and shape how (indeed, whether) one experiences the city as a *civitas*, and oneself as a citizen with entitlements and responsibilities. This *civitas* extends into multiple pasts (seen and used differently by different people), and forward by way of urban plans, visions and actions, and in the future's embodiment in urban infrastructures. In turn, citizenship can be threatened by erasure and amnesia, dispossession and fragmentation – and found again, or reinvented (see Arendt 1958: 198–9).

‘Street-level health work’ is exemplary civic work. Focusing on citizens’ bodies rather than material infrastructure, it is preoccupied with the continuity of life and the future well-being of the polis. Attending to the places and movements of public health research in an African city we discern their *civitas –* the ‘public’ in the notion of ‘public health’ – and attendant ‘geographies of responsibility’ (Massey 2004), which shape possibilities of public health and of civic engagement and political contestation.

My interest in contemporary science workers' encounter with the city and civic outlook arises from the experience of historical change. As a scientist studying worm infections, I first came to Kisumu twenty years ago. Since then, this provincial city – encompassing its inhabitants' lives – has changed from a sleepy backwater disconnected from flows of labour and commodities (fictions or aspirations), to a bustling hub of transnational non-governmental interventions and global science, sources of related expectations and learning, income generation and consumption. Having grown from 23,526 inhabitants in 1962 to approximately half a million today (Ogot and Ochieng’ 1995: 124), Kisumu is Kenya's third largest city. It is also an epicentre of the African HIV epidemic. This causes suffering, but, as discussed by Prince (this issue), HIV also contributes to new social forms and opportunities.

This personal experience of difference over two decades is complemented by the historical contrasts people in Kisumu frequently make between various pasts and presents when they talk about their lives, memories and aspirations, and when they move among the material structures of the city. The older generation contrast the time of their youth, the expansive moment of post-independence with its seemingly reliable structures and predictable futures, to the subsequent era of the long 1980s when economic and political decay, exacerbated by international terms of trade and policy directives, eroded their secure trajectories. Their children and grandchildren, born in the 1970s and 1980s, are preoccupied by a different contrast, beween that period of economic stagnation and political marginalization, during which they grew up, and a present marked by new global circulations, novel aspirations and imaginations – although the shadow of an earlier era of aborted hopes, before their own lifetimes, lingers on in their dream of the present.

Memories and remains of past cities fulfil a double role in this account. They are part of the ethnography, as traces of other cities endure in materials and memories into the present, referencing historical possibilities, unfulfilled hopes and past contradictions, and providing material resonance to contemporary city dwellers' struggles. And, as in other scholars' attempts to discern the shapes of the twenty-first-century city (see, for example, Caldeira 2001), these memories of a past, quintessentially modern city serve as a counterfoil and analytical comparison. Engaging patterns of historical contrast, this article about the place of scientific work in an African city begins in the past, in the first decades after Kenyan independence. These are accessed through old men's stories, told at the beginning of the twenty-first century in the now very quiet laboratories of the Kisumu station of the Kenyan Ministry of Health's Division of Vector Borne Diseases (DVBD) and in the rural homes of those who retired from there.^2^

## GOVERNMENT SCIENCE AT THE CENTRE: POST-INDEPENDENCE STREET-LEVEL HEALTH WORK

Between the 1940s and the 1980s the DVBD was the country's leading health research institution. Like other government bodies at the time (such as those for agriculture, livestock, wildlife, fisheries or water), it integrated scientific investigations and interventions, and collaborated with other ministries (see Ombongi 2010; Malowany *et al*. 2011). In the late 1960s Kisumu, one of the largest of over 50 DVBD stations (see Geissler 2011), employed 80 lifelong civil servants – cleaners and subordinates, technicians and technologists. Based in laboratories and offices in the city centre, neighbouring other healthcare and government institutions, DVBD workers conducted street-level fieldwork across the entire city and its environs. As they recall, ‘wherever government wanted to do something, we investigated’, or ‘wherever there was a problem, we were sent to research’ – ‘we were the eyes of government’, extending a panoptic gaze over the urban territory.

For urban malariology, for example, small experimental huts for night-time mosquito collection were distributed around the perimeter of the city and occupied by DVBD sleepers, incorporating scientific measurement into the built texture of the city. Regular malaria surveys had the entire city in view, visiting every house and backyard, examining semi-permanent huts as thoroughly as Asian traders' shop yards and administrators' front gardens – pursuing the ambitious aim of controlling insect movements across town, filing survey forms for every discovered mosquito breeding ground, and following up in correspondence with the site's owner.^3^ Movements between laboratory and field brought home specimens for analysis, but also went outwards, bringing new knowledge to bear upon the field: pedestrian spraying teams and environmental interventions targeted disease vectors in streets and homes around the city, and campaigns against endemic diseases and epidemics extended into the countryside using the station's Land Rovers and field stations.^4^ Pursuing a classic biopolitical vision of territorial coverage, they made the city known and knowable, expanding its scope.

Operating from a central governmental viewpoint, the public health workers experienced themselves as players in the making of a progressively healthier Kenya (see Geissler 2011). The uniforms they received (until 1982) embodied their double role as ‘men of government’: entitled to government provisions, and bestowing government care upon citizens. As a technologist who had worked in research from 1953 to 2003 remembered these bygone days of governmental identity:

In those days government workers had to have uniforms that they could be known by … brown shorts and shirts with a cross and an MD for Medical Department … . All departments had uniforms: veterinary, agriculture and medical. The uniforms later got finished. So nowadays you can't know … . You can think one is a government worker but he is just a mere person. (Retired technologist, 29 March 2001)

As government men, the DVBD staff were middlemen on clearly defined trajectories between centre and periphery that the young nation inherited from imperial geographies. All older men had spent years of their lives in the DVBD's headquarters in Nairobi and in various stations around the country. Encountering diverse pathogens, vectors and human populations, they performed valued work that brought the idea of the nation to life and shaped their identities as citizens and government men (see Geissler 2011). Their work was part of a collective effort of civil service, and the city they worked in and on was experienced as part of a national whole. Fieldwork was also nation building; the field was the polis.

### Living a post-colonial city

The geography of fieldwork as pulsating movements out of a clearly defined centre was reiterated in the men's personal biographies. With limited amounts of schooling, they did not belong to the city's elite – all were born and raised in rural ‘reserves’. They entered government service directly after school, choosing (Ministry of) ‘Health’ over alternatives like ‘Agriculture’ or ‘Police’ because of the high reputation of the medical profession (which was out of their reach). Moving from village to city, and ‘joining government’, workers up to the early 1980s were entitled to government housing in staff houses next to the laboratory, or, once they had family and gained promotion, in ‘estates’ on the formerly ‘African’ side of the city. These estates were premised on public health thinking, including piped water, sanitation and environmental control of disease vectors. Linked into the urban grid, they reproduced the aesthetics and functionality of post-colonial urban planning on an intimate scale: neat rows of identical houses with kitchen, living room and bedrooms, bathrooms with British cast-iron armatures, electricity connections and corrugated-iron roofing, small gardens and verandas complete with washing lines. Spaces between the houses were unfenced, tidied by (then functioning) municipal services. As the men pointed out when visiting these estates half a century later, ‘these places used to be so beautiful’. They shaped their inhabitants' understanding of well-being, provided a sense of belonging to the city and to the governmental project of the nation, and marked a trajectory of melioration – both for inhabitants, who saw their lives progress through improved housing; and for those who remained outside, in rural areas or informal settlements, for whom these estates held the promise of modern city life.

Projected around and after independence to make space for a growing, socially mobile African population, these estates were erected by municipalities, ministries and parastatals for their employees, and named after the entity that created them (‘Railway’, ‘Power’ and so on), or after nationalist heroes – Lumumba, Mboya, Ouko – who were associated with the progressive modernist project that the estates embodied. These heroes have long been murdered, infrastructures have decayed, water and electricity supplies have become erratic, and the link between profession and estate ruptured by subletting and privatization. Yet these estates still carry connotations of respectability and being part of a larger whole, and convey identities to their inhabitants, who often have lived there for generations.

This sense of belonging extended to the city as a whole. In fieldwork their governmental-medical authority gave the men access to every house; and as citizens, they remembered, they could walk freely and securely around town – ‘one could move anywhere, at any time … walking just home from cinema at night’ – and take their after-work drinks in spaces of public circulation, open to the street, like city-centre street bars or beer parlours in residential estates, or the different civil servants' clubs. Through these circulations on different levels of scale, as citizens and as agents of national government, they articulated civic space.

Their geographies of responsibility and entitlement entailed a progressive temporality, a vision of forward movement that would gradually encompass space. Their city was growing and getting better, driven by science and civic commitment; and at a distant point of convergence, the expansion of this *polis* would erase the centre–periphery gradients that marked their present, between metropolis and developing nation, capital and province, and between urban centres and rural reserves. The men experienced themselves as part of a vanguard on this trail of modernization – out in front, as men of science and government. As one retiree put it, referring to others in his home village: ‘in those days they hadn't entered the government. They were still backward … ignorant’ (Technologist, 18 December 2004).

Yet this did not imply that they considered themselves irrevocably ‘on the way’ from home or city towards more central places such as the capital city, say, or the metropolis of the former colonial power. Instead, their travels led them back to origin and family; after retirement they returned to their rural homes – either in their birth area or on bought land – where they had invested in modern houses, and established themselves as rural nodes of further modernist extension (entering local political or religious activism, opening clinics and laboratories alongside other business, promoting development). Lives and scientific work were situated within one concentric geography.

The old men's portrayal of ‘their times’ puts less emphasis on contradictions and tensions in the post-colonial project. The city left behind by colonial occupation was marked by violent ruptures – the men recalled, for example, a turnpike that separated ‘white’ and other areas of the city during the 1950s repression of anti-colonial resistance (which some had supported). But as in any modern city, these differences were imagined – notably by public health planners and workers – as a whole, connected by public spaces and services, and at least ideologically encompassed by an overarching process of modernization (see Caldeira 2001). Even colonial, racist urban planning had had a totality in view, with white zones of leadership, Asian zones of commerce, and (limited) spaces for African labour, mutually reliant upon one another.

Some men recalled conflicts within the DVBD after the British officers' departure – for example about the contentious practice of ‘human landing catches’ (a preferred method of capturing mosquito specimens in which the technician is simultaneously hunter and bait) – and arising out of the increasingly authoritarian government; yet these tensions were contained within an overarching commitment to civil service, to which most men belonged continuously from school age to retirement. Their Nestor thus emphasized the non-partisan nature of their civic engagement, à propos of post-colonial labour conflicts: ‘We were told that, being a civil servant, don't indulge yourself into politics’ (Retired technologist, 24 March 2001). A colleague, employed after independence, explained: ‘We could not mingle [party] politics with being a civil servant. We were not allowed … not only allowed, but whoever liked his work could not mingle it with politics' (Retired technologist, 30 May 2005). Politics here equalled particular interests, as he explained concerning the contested practice of human baits in mosquito research: ‘Our work was research and was hard; putting in politics would mean a lot of strikes, a lot of argument, for example about us being used as baits (for insects). You just had to tolerate things – if you would have mixed it with politics, DVBD would not have succeeded’ (Retired technologist 30 May 2005). Rather than by political interests, their civic vision was shaped by ‘discipline’ – a common term in conversations – proudly demonstrated by the men in morning parades and obedience to officers.

In turn, they expected from their fellow citizens that they would assent to examinations or treatments: ‘Nobody could refuse [blood samplings]. How can you refuse and the government wants to help you?’ Establishing consensus was part of fieldwork: ‘You have to convince people, when they agree with you well is when you can work. There has to be unity between the chief, the [health] workers and people, then work progresses well.’ And if necessary government force could be deployed: ‘a chief … knows the people in this area. … If he explains well to them they like it, so they just come. … But if someone refuses then we report and they are the ones who know how they can deal with them’ (Technician, 17 September 2004); ‘Some were resisting but later on they accepted after we had taken some of them to the to the police and they were arrested and given some canes’ (Retired technologist, 30 May 2005). As civil servants, the DVBD men represented a civic whole, which allowed little space for particular interests, and which moved forward guided by science.

Such overarching continuities and certainties are no longer taken for granted. Through decades of economic decline, growing corruption and structural adjustment, public health work diminished together with other government services. Decline reached DVBD with a slight delay, as external funding for time-limited research projects (like the one I worked for at the time) created a brief period of bounty for the DVBD men. Towards the end of the 1990s, however, this flow of equipment and *per diem*s dried up, as research collaborations grew in scale and shifted away to the parastatal research organization, KEMRI, as part of a new economy of health research and intervention. The DVBD station continues to exist, but, without a government operating budget, it has a much-reduced reach: with disconnected telephones, grounded vehicles and without laboratory supplies, the few remaining, highly qualified DVBD staff rely on infrequent short-term engagements with other research organizations; otherwise, they occasionally survey a school within pedestrian reach, using recycled microscope slides, to report epidemiological figures to their Ministry. They still do this, because they do not like ‘sitting idle’, and because, as a token of twenty-first-century change, they are now tied by ‘performance contracts’ that demand documented activities irrespective of lack of funding. In the meantime, they read up for their distance-based diploma courses, or wait for an occasional relative who needs a diagnostic malaria test. The wider circulations of which they were once a part have been disrupted; few scientific publications emanate today from (or reach) their labs, and their reports and requests to the Ministry are not always acted upon.

## GROWTH IN A DISEASED CITY

During the long 1980s, Kisumu stagnated: industries closed down and international goods traffic diminished. *The Lonely Planet* described Kisumu's ‘decaying atmosphere … a bit of a dead end these days … just marking time’ (Finlay and Crowther 1991). When I first lived there the only venues for dining out were a decaying ‘Wimpy’ and the terrace of the ageing colonial hotel, and the only expatriates were British or American volunteers descending upon town at the weekend. However, in the late 1990s the city experienced a recovery, based on service and leisure, consumption and transportation, and an exploding real estate market. By 1997, the *Lonely Planet* surmised that ‘Kisumu's fortunes may once again be on the rise’ (Finlay and Crowther 1997), noting a growing range of hotels at rising prices, and by 2000 and 2003, the guidebook could describe new shops, private clinics, hotels and guesthouses, coffee shops and pizzerias, live music venues and new cinemas showing the latest American movies (Crowther 2003).

A striking geographical shift is embodied in large enclosed malls, complete with supermarkets, clinics, lounges, open-air ice cream parlours and pizzerias modelled upon global fast-food chains, cinemas and fashion shops, all surrounded by walls and protected by private security guards, built around the millennium by local investors and international franchises. These are not in the planned city centre, where the colonial business district and central market were, but on the city's marginal lands, adjacent to slum areas. They exhibit commodities and foods that a decade earlier were unavailable (and which even today few can afford), and provide seemingly placeless locations in which patrons may socialize (if only infrequently owing to high costs), sheltered from the hassle of the growing, disordered city. For many others, the malls' high walls, over which rise large billboards and neon adverts, contain what the world has on offer, but to which they have no access.

With these new forms of exclusion, new connections – available to some – appear. While the railways were privatized and stopped working, air transport multiplied, culminating in a nominally ‘international’ airport inaugurated in 2011; cybercafés expanded virtual connectivity at plummeting rates; local transport, which in 1990 was based on battered public buses and Peugeot 404s, was changed by thousands of bicycle taxis, followed by motorcycles and motor-rickshaws (reflecting Chinese trade influence, and the operators' rising fortunes), and a growing number of minibuses and private cars choking the roads.

Prices for housing and land have skyrocketed. In 1993, a colonial bungalow in the former ‘white’ area could be rented for KSh10,000 (about £90) per month; the same building (revamped and securitized) today commands KSh100,000 (about £900); a mud-walled and iron-roofed room in an informal settlement, then less than KSh100, may cost up to KSh1000 today. Following demand for security and reliable power and water supplies, ‘gated’ housing, like the malls often situated outside the planned city in semi-rural wastelands, have become the model of real estate development. The expanding market in housing-for-rent and plots for construction – associated with (salaried) ‘working-class' investment strategies – has multiplied estate agents and increased speculative investment. Expensive short-term rental facilities cater for expatriate workers in HIV NGOs and research, and pricy ‘homestays’ for younger expatriate volunteers’ needs. These urban developments converge in as-yet-unbuilt real estate dreamscapes such as ‘Railway City’ on the lands of the privatized Kenya Railways. With its proposed luxury hotels, high-rise apartment blocks and floating restaurant – Dubai style – this vision entails not the further development of the existing Kisumu, but an altogether different urban future that turns its back to the city, cutting it off from the lakeside.^5^

The growth of urban condominiums and malls has been accompanied by the expansion of slums (UN Habitat 2005). This reflects widening economic disparities, and the formation of new economic classes, including a ‘new middle class’, which some see as an indication of economic and social progress (see, for example, African Development Bank 2011).^6^ Much of Kisumu's population experiences growing levels of poverty: rents, food and water prices, power and fuel costs are rising continuously, while the lowest incomes have hardly changed (daily payments of less than 100 shillings are common for casual employment, which is still hard to get).

Changing class relations loosen urban attachments: municipal workers and government employees, who previously had been entitled to government housing in centrally located estates, have lost this position in the city. The new boundaries around malls and housing estates, protected by barbed wire and a burgeoning private-security industry, have changed conceptions and uses of public space, fragmenting the tissue of the city and weakening its original centralized structure around a public core. At the same time, the walls that now shape Kisumu lend themselves to projections of urban desire and dreams of escape.

### The city as experimental social space

These developments occurred in parallel with the HIV epidemic in Kisumu. As few other major economic activities have developed during the same period, HIV interventions seem linked to economic change (here, economic studies would be valuable). Kisumu has become an ‘NGO city’ (see Prince 2012) where non-governmental entities drawing on international (mainly US) HIV funds, are main employers and principal forms of entrepreneurship. HIV-related activities employ tens of thousands,^7^ and provide opportunities and income to most of the city's working population, including service industries and transport. HIV funding has brought expatriate experts and volunteers who today are a visible population group, powerful consumers and lifestyle models.

HIV-related research and interventions operate in all health facilities. Research often focuses on poorer populations heavily affected by HIV, or high-risk population groups – ‘sex workers’, young people. In addition to clinical trials – that is, ‘experiments’ *sensu stricto –* the large-scale innovative HIV interventions are also ‘experimental’ in that they are knowledge-generating practices that operate in a spatially and temporally circumscribed fashion (see Nguyen 2009). The new urban shapes and circulations, and people's attempts to live meaningful lives upon this shifting terrain, are thus intimately intertwined with bioscientific research and intervention. Moreover, the city is ‘experimental’ not just in the sense of hosting important research, but also by virtue of the novel economic opportunities and social forms that medical interventions and research have brought. Today's scientific workers exemplify a social group undergoing expansion and change in this context.

## SCIENCE WORKERS, EARLY 2000s

### The Kenyan Medical Research Institute (KEMRI)

Following a pattern now familiar elsewhere in Africa, scientific work in Kisumu today relies on ‘collaborations’ between local parastatal and non-governmental institutions and ‘Northern’ agencies (see Okwaro and Geissler, under review). KEMRI is a main partner for transnational research and intervention, collaborating with major UK and US universities and funders. The US government Centers for Disease Control and Prevention (CDC) have worked through KEMRI for 34 years, and in 2008 engaged about 1,200 staff and many more research participants on an annual budget of approximately US$25 million. The total funds disbursed through this collaboration are considerably higher, including expatriate staff and visitors salaries and allowances, and project resources from other funders. This makes the CDC funded KEMRI/CDC programmes the biggest single employer (apart from the civil service) in the city and the largest source of income and monetary circulation.^8^

Geographically, this public health collaboration reiterates some of the spatial changes described above. Its main sites are scientific enclosures: well-organized and highly productive spaces that allow for clinical and laboratory work of globally certified standards. They are separate from the city's circulations, necessarily protected from the surroundings on account of their technical and material resources and the surrounding lack of these. The main ‘field station’ is literally outside town; not linked to public transport, it is reached by private cars or the KEMRI/CDC shuttle bus; its power and water supplies do not rely upon the municipal grid with its erratic supplies, and its own satellite connections allow real-time communication with the CDC headquarters and other global centres of excellence. Kenyan staff therefore sometimes jokingly called the station ‘Atlanta’ – as much a local instantiation of global power, resources and knowledge as a place in Africa. The other main CDC research site, the ‘clinical research centre’ on the grounds of the provincial hospital, is less obviously out of Africa, but its modern offices and landscaped gardens, too, stand in aesthetic contrast to the 1960s concrete of the neighbouring hospital buildings. Like the field station, it must be protected on account of the technological and medicinal resources it contains, and resource transfers between the research centre and the surrounding, resource-poor territory are inevitably limited.

‘Centres’ like this are, unlike the old DVBD laboratory, not central nodes of local government and of circulation – of people, drugs, information, and administrative power – that integrate civic territory. The mid-twentieth-century post-colonial territoriality represented by DVBD aspired (if often in vain) to expanding urban order, panoptically knowing and progressively transforming the city from a central administrative-cum-scientific locus (see Packard 1990; Vaughan 1991; Arnold 1993; Tilley 2011). Today's resource-rich enclosures, by contrast, resemble islands upon which modernity, scientific truth making and medical capacities have coalesced. The relation of researched spaces to the totality – city, nation, society – is here one of sample to frame, rather than of centre to whole. This changes the knowledge produced by scientific fieldwork: it operates here not as a simple vector along which action is directed back to a specific site, group or health problem – as when DVBD investigations directly extended into control campaigns – but as representative ‘data’ from selected individuals and sites, from which one extrapolates generalizable knowledge that takes effect not where it came from, but in the spheres of international science and global policy formulation.

As far as public health is concerned, there is no longer one ‘modern’ city comprised of differentiated parts integrated by a shared ‘public’ grid and circulation, of which health research was part along with municipal bureaucracy and civic infrastructures; no longer one project (be it colonization or nation building), but an archipelago of public health (Geissler 2013). So how does one move and live within these novel urban formations?

### Twenty-first-century public health researchers

We can trace changes to city lives and civic imagination through the local workers of the KEMRI/CDC research collaboration: doctors and nurses, fieldworkers, counsellors and follow-up staff, drivers and technicians employed on short-term contracts funded by grants. For this study, we focused in particular on the staff of one large urban HIV prevention trial; while their tasks were specific and their field very different from, for example, rural village reporters (see Chantler *et al*. 2013), their demographic, social and professional profile resembled that of other local research staff employed by the collaboration.

Most of them are between their mid-twenties and forties. Many are women, some of them breadwinners or single family heads – a radical shift compared to the all-male DVBD research staff. Generationally, they are children and grandchildren of the DVBD men (indeed, some descended from them), who, with their old laboratory in the city centre, remain a familiar fixture, an ancestral group, and a foil of contrast. When they meet, such as when DVBD staff assist in KEMRI/CDC projects, or when KEMRI/CDC staff visited us at our office at DVBD, their relationship is marked by generational respect, but also by tensions arising from differential access to resources, and different styles of work and comportment.

Born between late 1960s and early1980s, most of these KEMRI/CDC staff had known fathers in regular employment, usually with government: teachers and municipal, health or agricultural extension workers, survey or laboratory assistants, and the occasional nurse, clinical officer or doctor. Some mothers had been employed as teachers or ran small businesses. Accordingly, most had grown up in urban government estates, moving between Kenya's main cities as their fathers transferred between postings, and most spoke a local language as well as Kiswahili and English. Their schooling exceeded that of their parents (and of the DVBD men), and included fee-paying secondary and tertiary education, together with socially similar children from around Kenya. By contrast to the DVBD men, however, their peregrinations around the nation coincided with economic and political decay; rather than knowing the excitement of nation building, they came of age with mounting doubts about their country, witnessed the urban government estates falling into disrepair, and suffered the eventually loss of their parents' entitlements to live there.

### (Self-)Discipline

Most of them had endured declining family fortunes due to the devaluation of government work, and of education. Many were orphaned in the early years of the AIDS epidemic and completed their schooling with the help of older siblings. Discipline, so important a topic for the ‘nation builders’, was also a prominent theme in conversations with these younger people. It featured both in relation to their upbringing – most parents were described as strong, mainstream Christians who valued discipline – and in speaking about work and career. This may reflect the pressure of self-representation in a workplace situation, but there is little doubt that for these young people making headway under present conditions requires determination. They praised virtues like punctuality, long hours, systematic routines and neat dress, again topics dear to their DVBD forebears. Yet, while the old men's discipline seemed associated with duties towards a collective, and obedience to governmental hierarchies, the young KEMRI/CDC staff were less subservient, nor did they invoke an ethics of service or the wish to build a healthy nation.

None expressed the desire to work for Kenyan government institutions despite their anxiety associated with short-term contracts, and almost all dreamt instead of employment with international organizations, preferably as expatriates abroad, seeking to escape from what they perceived as a society eroded by lack of resources and opportunity, self-interest and corruption (see also Redfield 2012). One particularly critical study manager summarized issues others had raised in less pointed language:

I hated government jobs. … One, they are underpaying. Two, people don't work. Three, you don't have a conducive working environment. Four, you are underutilized … and the other thing is that it's like there … there are lots of old people there who are very sluggish in doing their work. … Initially I thought of being a district officer … but then I looked at the … the theory of goats being given to somebody who is a DC, you are being given hand-outs and I thought no, that's not my life. (Research coordinator, 30 May 2007)

Rather than as the labours of government, they saw their work as part of ‘global health’; and as good, satisfying and well-paid work, a move up from the jobs with NGOs and commercial bodies that they had had earlier. Accordingly, they focused on getting their work done efficiently and well; which for the old men had been synonymous with hardship, investigations into the unknown, and making tangible improvements, was for them about validity, thoroughness and adherence to standards: ‘research is very strict in the ways that they do things. So, actually it builds discipline in you; that this is how research is done. And I think I love that’ (Research nurse, 28 January 2009).

### Service

Yet, although seemingly focused on individual careers, training and remuneration, and contemplating personal ‘exit strategies’ from city and country, these urban staff frequently expressed regret at the present state of public health, of their city (and of their country's government); they also expressed a longing to contribute to health in Kenya. KEMRI/CDC engages in research and intervention; but while, for DVBD, public health and disease control were primary, and research and data circulation were tributary tasks, KEMRI/CDC staff are employed to conduct research, and their public health role is at best indirect, through publications and policy.^9^

Instead of associating work itself with duties to a collective, the research workers' ‘right to give’ (and their search for larger collective forms) was articulated partly around employer-led voluntary schemes – medical camps for displaced people, AIDS day fund-raising activities, blood donation runs, school health education – and in private gestures towards individual poor research participants, as well as in the satisfaction derived from being able to help patients with research resources: ‘When I see the babies I follow are very healthy and don't turn HIV-positive – they don't die – I feel encouraged and I feel so happy and just call myself successful’ (HIV counsellor, 12 December 2008); ‘Even after work, when you see [research participants] outside doing quite well you get … that satisfaction’ (Junior doctor, 14 March 2009). While young fieldworkers who had grown up under the shadow of AIDS enjoyed spreading newly acquired knowledge to age mates, doctors enjoyed their ability to achieve clinical results:

If we have a patient and a drug is not available, I send for it and [the collaborators] bring it. But in [the government hospital] we just write a prescription; but do you really care about the patient at the end, because you give the prescription and the patient cannot afford it, but you have done our work by writing the prescription? So there is no job satisfaction there. (Senior doctor, 27 April 2008)

The idea of serving and being part of a larger whole persisted (Tousignant, this issue; see also Wendland 2010), but civic desires focused on particular individuals and situations and tend to be perceived as separate from work. Sometimes they may even infringe work ethics and rules, as when staff give participants small gifts, unsure whether this contravenes research ethics regulations against payment and ‘undue inducement’; or when clinicians continue to provide advice, diagnosis, and sometimes treatment, to participants who have finished a trial and formally should no longer be contacted or treated, but to whom the doctors feel clinically committed. Public and private, civic and professional commitments are no longer simply aligned in a taken-for-granted ethos of civil service.

### Collectives

More than for their parents, who after training in colonial schools gained lifelong employment among ‘government men’, for the young generation life seems a lonely struggle. Many described an everyday comprised of work, business activities and family, with little time or money for socializing. Some mentioned that their employment in a well-known organization believed to pay high salaries had strained their personal friendships and kin relations; others described how social ties with new colleagues and peers were limited, partly due to competition: ‘Everywhere you go, you find [colleagues]. I am … careful about mixing my social and professional friends, so all the friendships I make at the workplace, they end at the gate, so outside there, I pick a new social life’ (Nurse, 22 October 2008). This contrasted with the DVBD generation's lifelong kinship-like bonds with workmates, and shared social lives (Geissler 2011). The main social association for most KEMRI/CDC staff remains Church – often in more recent evangelical and Pentecostal movements. And many, especially female staff, were members of (women's) groups and merry-go-rounds, which combine saving and investment with social purpose.

KEMRI/CDC salaries and allowances are in the upper ranges of Kisumu's emergent middle or, in local parlance, ‘working class’.^10^ While temporary and dependent on external agencies, they exceed the wages of DVBD men or equivalent health professionals in public hospitals. KEMRI/CDC workers also consider themselves as of a particular class because of their wider knowledge and networks, and they have a marked identity as ‘being with’ KEMRI/CDC,^11^ contrasting this to working ‘for government’. When asked to describe the organization they work for, many located funding and decision making in Atlanta, USA, positioning their work not in the nation but in global (health) space, in which they are well-connected, but also marginal and dependent.

Their sense of organizational attachment resembles that of the DVBD men. However, for the old ‘men of government’ the nation state had provided stability and nation-wide mobility. Despite colonial tensions and post-colonial political alienation, their identity referenced a localized collective. By contrast, attachment to KEMRI/CDC does not convey a straightforward identity or belonging; instead workers must, along with research work, invest continuously in shifting local institutional and personal ties in order to stay connected in the city's ephemeral public health economy, and to open up diverse alternative options (see Prince 2013 on the moral economy of HIV).

### Researching the city

Most urban research participants, like the women participating in the HIV prevention trial that we studied, live in informal areas that surround the city like a belt, between one and two kilometres wide. These areas are much more diverse than the label ‘slum’ would suggest, but on average they are poorer than the planned central urban areas. Participants paid monthly rents of between 500 and 2,000 shillings for semi-permanent, often one-roomed houses without gardens or courtyards. Driven by poverty and strained social relations, they moved frequently between different places in city and countryside, including their birth home, marital home and the houses of other relatives. In the absence of piped water and garbage collection, and in the presence of open sewers under conditions of extreme crowding, malnutrition and poverty, diseases are common. Participants are recruited here because of the higher prevalence of infections, especially HIV, and because the inhabitants of these zones, mostly unemployed or engaged in small-scale trade without access to medical insurance, are attracted to medical research with its healthcare benefits. The field is thus not ‘the city’ but circumscribed sections of it. Daily flows of scientific work connect two ‘extra-civic’ places, research centre and field.^12^

Although adjacent, the research centre and its urban recruitment areas are distant in terms of municipal and medical amenities. The perception of the field as a space apart is underlined by describing one's work ‘out there’ as ‘fieldwork’, ‘going out’ to ‘the community’.^13^ The latter term, which became important in the idiom of African medical research after the 1980s (Chantler 2012), references village communities with the implication of relative poverty and basic education, and would not be used for the inhabitants of the structured residential areas of the city, or ‘working-class’ people like the scientific workers themselves. Conducting fieldwork in the informal areas is associated with perceived risks, which underscore the sense of separation inherent to research work (although staff, off-duty, would probably move there without fear, at least during the day). ‘Study areas’ are here conceived of as spaces of data gathering, indirectly connected to civic, public health responsibilities through ‘global health’ policy. Fieldwork, then, is not primarily about transforming the field, but about generating generalizable knowledge that can travel elsewhere.

The geography of fieldwork is placed in relief by the ‘follow-up staff’ unit of the HIV prevention study, which we observed closely, a group of young female ‘community technologists’ led by a nurse. Attached to a trial, they visit research participants and their families at home in order to recruit participants, remind them of outstanding clinic visits, collect specimens, or check health conditions and drug intake. Regularly updated ‘locator forms’ – itineraries including bus directions, landmarks, phone numbers and people to ask for directions (or to avoid) – help them to find participants. Confidentiality is important in urban (especially HIV) research, and workers often present themselves as health workers, or church friends or relatives rather than researchers (Madiega *et al*. 2013), which simultaneously opens space for more personal engagements and adds distance from the field. Fieldwork often involves issues like sexuality or childcare, feeding or hygiene, HIV disclosure and domestic conflict. It means entering other people's lives, spending time, encountering participants and their neighbours and relatives. The resulting personal ties and commitments can be, as described, personally satisfying, but also potentially economically taxing and ethically complex.

Skilful negotiation is also required of follow-up staff when they enter government health facilities, such as when following sick participants admitted to the public hospital. Bringing additional medicines that are unavailable in the hospital pharmacy, or food and utensils, and taking measurements and observations necessary for clinical care or for research, which overcommitted government staff did not collect, research workers combine detailed knowledge of procedures and shortcomings of the government system, and research expertise and resources, as well as diplomatic skill to bridge the gap between transnational clinical trials and local medical realities, supporting patients with the least possible disturbance of hospital routines.

The daily making of scientific knowledge requires from research staff careful linking of different situations and places in order to generate answers to specific questions. The city features here as a heterogeneous association of actors and relations rather than a civic whole. The residential patterns of urban science workers provide some additional insights about their changing place in the city.

### Science workers' home in the city

The houses of expatriate scientists and local participants project the epistemological distinction between researchers and researched, as well as the realities of global inequality onto the city map: expatriate scientists reside predictably in the better-off parts of town, the erstwhile administrative quarters, either in a gated compound or in converted former colonial bungalows with back-up power supply and water filtering systems; research participants live, as mentioned, mostly in the informal ‘slum’ belt around the actual city. This polar distribution is as unsurprising as it would be hard to avoid, reflecting the distribution of financial resources, medical problems and expertise. Local research workers, however, complicate this simple geography.

Local employees on temporary contracts probably have more in common socially with their participants than with expatriate scientists.^14^ Accordingly, one may surmise that research workers share participants' spaces, on the poorer, informal, and, in colonial terms, ‘African’ side of town. However, when we plotted KEMRI/CDC staff's houses on the map, these were not in zones of urban neglect, except for small enclaves of more expensive housing, enclosed by private compounds. Neither did they live in the planned residential estates of colonial origin; a few senior clinicians lived in apartments in the formerly ‘Asian’ high- class area close to the hospital, but none in the colonial ‘white’, administrative zone; and very few, staying with parents or husbands, lived in the more central, modernist government housing estates of the 1960s and 1970s. Most lived instead around Migosi, an inconspicuous sector of the belt of housing outside the original planned city – but, unlike the rest of this belt, not designated as ‘slum’ by UN Habitat.

That this differentiation surprised me exposes my ignorance; no citizen of Kisumu would have conflated Migosi with adjacent ‘slum’ areas; in Kisumu estate agents' lingo, it is an ‘up and coming’, ‘very attractive middle-income location with very good rental prospects’, drawing in investors and developers. Between city and countryside, this is a transitory territory in more than one sense. It is not entirely formal, as many residents have no land titles or construction permits, and as the area has not been integrated properly into the municipal development plan, water and electricity supply, and road net. But neither is it ‘informal’ in a derogatory sense; most houses are built from cement blocks, increasingly many are multi-storey and freshly painted, displaying ‘luxury’ features like balconies, tiled roofs and columns, while some have (small) walled and gated ‘compounds’ that fetch a premium on the rental market. With many shops and guesthouses, hooting minibuses, swarms of bicycle taxis, and trendy young people on the street, Migosi has a feel that contrasts with the old governmental estates and their decades-old rental arrangements. Although not a planned ‘estate’, Migosi vigorously projects a shared identity, including citizen associations and self-help groups, and a small business community with a website.

Living in Migosi means to be in-between socially, threatened by descent into the poverty of the slums, and hoping for home ownership in the new suburbs. Also, in terms of leisure and consumption, those living in Migosi moved continuously between spaces. For busy working-class people, the supermarkets in the major shopping malls, built since 2000, allow for convenient and safe after-work shopping (at least for some commodities), with motor rickshaw and bicycle taxis departing from the protected gates. Within these enclosed malls, younger professionals find fashionable clubs and cafes where one can sit outside, even at night, and meet people of ‘the same class’, undisturbed by traffic and exhaust fumes, beggars or potential thieves.

The old DVBD men, by contrast, recalled 1960s leisure activities that were on hand as one walked around in the city's central venues – shopping street, cinema, dance halls – and continued for decades to patronize the same roadside ‘joints’ or ‘pubs’ for after-work beers, or simply went to neighbours' millet beer parties. Their grandchildren in health science prefer, within a changed city, more exclusive and fashionable venues, that reference the placeless aesthetics of global connection. While these places, and the commodities they extend, may be considered ‘modern’ compared to the rest of Kisumu, this is a different sense of modernity from that implied in memories of the 1960s. The city's centre was once imagined as a hub from which modernity expanded. By contrast, the shopping mall modernity is a space apart; to become modern means entering confined spaces, escaping from the shortcomings of the surrounding territory (see Ferguson 2006). If this modernity still points to a future, this future is elsewhere.

Among young urban professionals, Migosi at the time was considered the most attractive of the realistically achievable areas of town. When asked why they lived in this area, KEMRI/CDC workers cited, apart from just-about-affordable rents, that there was ‘good security’, because of the relative social cohesion and public circulation, even at night; and they praised convenient transport and shopping facilities, good (private) schools, and the availability of water from traders. Living in Migosi combines flexibility, convenience and safety, and although rents have increased during recent years, suitable flats for small families and single tenants are hard to get. Tenancy contracts are passed on along lines of friendship, or sublet.

The fact that most houses here are for rent makes Migosi particularly suitable for workers with transnational and non-governmental organizations. On temporary contracts, they dispose of relatively high incomes, but have no mortgage securities. A house of one's own remains their aim, but building is done in small steps: saving to buy a plot, erecting small constructions to mark ownership, cementing the foundation, and buying building materials according to one's income situation. This process takes years, and few staff actually own a house. In the long meantime, they rent, moving into better housing after a pay rise, or moving the opposite way after retrenchment or during contract gaps. The stable lives of their forebears in public health, half a century earlier, who ‘joined government’, married and raised their children in government houses, and retired to their birth homes, are a distant memory.

### Remembering futures and pasts

A well-kept memory, though: the old municipal, council and parastatal estates still feature in the KEMRI/CDC workers' thoughts and aspirations. They appreciated the beauty and solidity of their lasting structures, embodying past comforts and certainties, collectives and projects. While other urban structures – like the 1950s railway station and its overgrown tracks – were now merely sites of memory, triggering nostalgia without hope or desire of return, modernist housing estates remained attractive also for the young, even at their high sub-let rates. This is because of materials and location, and more reliable access to water and the power grid, but also because of aesthetic qualities, such as regularity and developed gardens, and the stability provided by long-term residents and older civil servant families as neighbours. Even among this young and dynamic cadre of scientific workers, then, the past modernist spaces that had impressed hopes for the future and civic virtues upon their grandparents remain coveted, although they are – together with everything they stood for – hard to reach.

Just like these sites of past futures, the places of past origins, the rural homes, which had constituted the second fixed point and destination of return in the older civil servants' lives, are not within easy reach of the young science workers. Many of them – in particular women (often breadwinners) – have neither access to land (something that earlier generations, including the DVBD men, enjoyed) nor the obligation to build a customary home. Some men build houses in a rural home, but many younger men no longer have an unequivocal place of origin and return. Due to patrilineal kinship complications arising from the parents' early deaths, remarriages and urban upbringing, few inherit uncontested land, and land shortage and rising prices exclude even young professionals from land ownership. Many postpone customary home making and focus on an urban house, or real estate investments such as rental rooms or shops. Those who do build houses, however, are often at pains to make reference to the rural past by adhering to customary building and ritual practices (Geissler and Prince 2010).

The countryside now features as investment rather than as ‘home’: those who are not (yet) able to buy a rural ‘farm’ in a cash-crop-yielding rural development scheme invest disposable income in shorter-term agricultural leases for sugar, rice or maize production. Such investments, like acquisition of farmland, are usually not connected to one's original home area, to avoid contradictions between capital accumulation and kinship obligation. Where professional lives once were situated in predictable circulations between lasting rural and urban spaces, today, connections between these areas are temporary and speculative.

Life in Migosi has more future than past.^15^ It looks forward, but this future is different from that of 1960s modernist urban estates, which seemed to be of one cast, made up of concentric rings – estate, city, nation. This simple vision of colonizing civic space, expanding and improving with water pipes, electricity wires and roads – and assisted by public health officers – has been replaced by a less predictable interplay between individual needs and initiative, opportunity and limitations. The futures of today's science workers cannot be assembled into one coherent map and schedule; yet, although unstable and ephemeral, they are also less finite, more open to dreams and speculations.

## END: CIVIC FUTURES IN-BETWEEN AND ON-THE-MOVE

This article described the place and movements of public health workers in a changing city. Compared to the1960s modernist city described by a cohort of now retired old men, contemporary public health workers' geography appears marked by fragmentation and enclosure, ruptures between past and present, and between different spaces within the city – an archipelago of modern islands, without the redeeming fiction of a shared *civitas*, nor the hopeful narrative of modernization that turned the post-colonial cityscape into a possible space of appearance.

This portrayal of the present resonates with scholarly descriptions of millennial urbanity as opposed to the modern city, as marked by new segregations and boundaries, by shrinking public spaces and circulations (Harvey 2006; Ong 2006; Caldeira 2001; for Africa, Mbembe and Nuttal 2008; Murray 2010), and connection to widening grids across distance and scale (Sassen 2006). Larger flows of expertise, power and resources overwrite the city, and changed urban landscapes reshape citizens' relations to larger collectives, political directionalities and responsibilities (Massey 2007).

This simple contrast between modern and millennial city is qualified, especially in Africa, by the persistence of colonial and post-colonial spatial forms and historical inequalities: Kisumu was not a built expression of egalitarian enlightenment but of colonial exclusions; and, by contrast with wealthier cities in the North, traces of this violence were not erased by renovating and reconnecting the urban texture.

Qualifying simplified historical contrasts further, Africanist ethnographers often observe localized improvisation, creative reordering upon ruined territories, ‘making do’ in post-neoliberal end-times, pragmatically navigating a landscape of ruins and novel boundaries. Here, historical contrasts become part of contemporary sociality, mixing progressive aspirations with nostalgia for past hopes and aspirations (see, for example, Hansen and Vaa 2004; Simone 2004; Roitman 2008; De Boeck 2011).

Similarly, our scientific workers move and find their place within the decomposing *polis*, between enclosures and exclusions, and between various alternative, partial civic affiliations – temporary employment in global health, ethnic affiliations, NGO work, HIV self-help associations or community groups. Some long for a past urban life that is ambiguous and only partially accessible through its remains, exemplified by respectable mid-twentieth-century urban housing; others dream of futures beyond their city, in the global world cities that are as speculative as the fictions of real estate developers.

Unable (and probably unwilling) to return to the lives and civic imaginaries of their fathers (apart from ‘cultural’ gestures), and as yet unable to transform their present into a better future, twenty-first-century Kenyan public health workers find themselves in-between, geographically, economically, and temporally. They are a very different ‘middle’ class from their nineteenth-century European namesakes or their post-colonial ancestors: not quite here nor there, longing for unreachable futures and pasts, suspended in the present, at risk of dropping out. Staying in the middle requires persistence and flexibility – as witnessed by the slow-growing foundations of the science workers' dream houses. Sometimes they feel stuck and dependent, at others up-and-coming. Maybe this new, ephemeral constellation, free of the modernizing telos and faith in one's city and nation, will produce new political imaginaries, modes of association and narratives of transformation. Although familiar bonds between urban landscape, commitments to others and dreams for the future have been ruptured, other geographies of responsibility may yet take shape.

## Footnotes

1 For Arendt the polis is not merely ‘the city-state in its physical location’ but the ‘organization of the people as it arises out of acting and speaking together’, that is, a ‘space of appearance’ (1958: 198). This polis relies upon the intentional work of those united by it; it ‘does not survive the actuality of the movement which brought it into being, but disappears not only with the dispersal of men … but with the disappearance or arrest of the activities themselves. Wherever people gather together, it is potentially there, but only potentially, not necessarily and not forever’ (*Ibid.*: 198–9).

2 This material stems from an ethnographic-cum-historical study on which the author worked with Kenneth Ombongi, Maureen Malowany, Philister Madiega and Ruth Prince.

3 Kakamega Records Centre DA/22/1, PC Nyanza, Medical Services, General correspondence, 31.12.1951–20.12.1963.

4 Kakamega Records Centre ND/12/34, Medical Department Annual Reports, Kisumu/Siaya 1953–77.

5 See <http://www.krc.co.ke/joomla/images/Internationaltender/KSMRAILWAYCITY.pdf>, accessed 2 June 2013.

6 Whether this middle class indeed is an industrious, value-creating bourgeoisie remains to be seen. The use of the term ‘class’ is open to discussion: while Kisumu's current economic boom is reminiscent of what older Marxist analysis described as ‘rentier capitalism’ – premised upon the circulation of capital unattached to production – the beneficiaries of floating capital in this case do not own its sources. Their status is a matter of consumption and lifestyle, not of property. The HIV economy resembles the ‘rentier state’ described for nations relying upon natural resource income, with similar implications for the development of civil society and democracy (Omeje 2008).

7 Actual numbers are hard to calculate because of informal labour arrangements and sub-contracts, including so-called ‘volunteers’, who support HIV-related activities without contracts in return for small, often irregular payments (for example, clinical trial volunteers, HIV-positive ‘peer counsellors’, support group members, HIV clinic helpers, NGO volunteers) (Prince 2013).

8 This section draws on ethnographic research, jointly undertaken with Philister Madiega and Gemma Jones, on the KEMRI/CDC collaboration, in the course of which almost 100 in-depth interviews with staff members were conducted in 2007–9.

9 That ‘research-to-policy’ is a less than straightforward linkage is evidenced by the burgeoning literature on this (novel) challenge (see, for example, Gilson and McIntyre 2008); the policy-to-practice link, on the other hand, is hampered by the decay of health systems.

10 The least qualified earned almost KSh20,000 KSh/month (about £170) in 2009 and experienced laboratory technicians or fieldworkers up to KSh50,000 or 60,000 (£400–500). By comparison, research participants pay monthly rents between KSh500 and KSh1,000 and many have difficulties securing KSh50 or so for a basic meal with their children.

11 Although their temporary contracts are with KEMRI, their salaries are paid from funds transferred annually by CDC.

12 In a similar fashion, the rural ‘Health and Demographic Surveillance Area’, inhabited by 100,000s of people, is a clearly demarcated zone of investigation and intervention, while adjacent rural areas are much less affected by research and the healthcare that comes with it.

13 On the propensity of medical research (on HIV in Kenya) to map out new geographical and social spaces and thereby create new spaces and social categories, see, for example, Booth 2004: 86, 94, 127.

14 This observation runs counter to a foundational distinction between ‘researchers’ –irrespective of their employment status – and ‘participants’, which allocates different positions and interests to the two sides – in relation, for example, to bioethics and the politics of public health research. The observation that epistemological and geographical sides do not necessarily coincide, and that Kenyan staff socially, and in terms of their contribution to scientific production and interests, might be closer to research participants than to expatriate global scientists, opens up a number of political and ethical questions (see Okwaro and Geissler, under review).

15 Or so it seemed at the time of fieldwork. As this article nears completion, Migosi's fortunes seem set to turn again. Underlining the ephemerality of contemporary urban order, Kisumu's main property website, under the title ‘Migosi falls from grace as rent skyrockets’ details how ‘Kisumu city's undisputed top middle-class residential district for the last two decades’ was becoming less popular on account of excessive rents and poor services. <http://kisumupropertymarket.com/migosi-falls-from-grace-as-rent-skyrockets/>, accessed 14 February 2013.
